# Super-Refractory Status Epilepticus: Prognosis and Recent Advances in Management

**DOI:** 10.14336/AD.2021.0302

**Published:** 2021-07-01

**Authors:** Batool F Kirmani, Katherine Au, Lena Ayari, Marita John, Padmashri Shetty, Robert J Delorenzo

**Affiliations:** ^1^Texas A&M University College of Medicine, College Station, TX, USA.; ^2^George Washington University, School of Medicine & Health Sciences, Washington DC, USA.; ^3^Epilepsy and Functional Neurosurgery Program, Department of Neurology, CHI St. Joseph Health, Bryan, TX, USA.; ^4^M. S. Ramaiah Medical College, M. S. Ramaiah Nagar, Bengaluru, Karnataka, India.; ^5^Department of Neurology, Virginia Commonwealth University School of Medicine, Richmond, VA

**Keywords:** super-refractory status epilepticus (SRSE), new onset refractory status epilepticus (NORSE), neurological emergency, status epilepticus (SE), and neuronal injury

## Abstract

Super-refractory status epilepticus (SRSE) is a life-threatening neurological emergency with high morbidity and mortality. It is defined as “status epilepticus (SE) that continues or recurs 24 hours or more after the onset of anesthesia, including those cases in which SE recurs on the reduction or withdrawal of anesthesia.” This condition is resistant to normal protocols used in the treatment of status epilepticus and exposes patients to increased risks of neuronal death, neuronal injury, and disruption of neuronal networks if not treated in a timely manner. It is mainly seen in patients with severe acute onset brain injury or presentation of new-onset refractory status epilepticus (NORSE). The mortality, neurological deficits, and functional impairments are significant depending on the duration of status epilepticus and the resultant brain damage. Research is underway to find the cure for this devastating neurological condition. In this review, we will discuss the wide range of therapies used in the management of SRSE, provide suggestions regarding its treatment, and comment on future directions. The therapies evaluated include traditional and alternative anesthetic agents with antiepileptic agents. The other emerging therapies include hypothermia, steroids, immunosuppressive agents, electrical and magnetic stimulation therapies, emergent respective epilepsy surgery, the ketogenic diet, pyridoxine infusion, cerebrospinal fluid drainage, and magnesium infusion. To date, there is a lack of robust published data regarding the safety and effectiveness of various therapies, and there continues to be a need for large randomized multicenter trials comparing newer therapies to treat this refractory condition.

Status epilepticus (SE) is a serious neurological emergency associated with significant morbidity and mortality [1-3]. As reported in a meta-analysis conducted by Lv et al., SE has a global annual incidence rate of approximately 12.6 per 100,000 people with no significant difference of incidence in males versus females [2]. The International League Against Epilepsy states that SE results either from the failure of the mechanisms responsible for seizure termination, or from the initiation of mechanisms which lead to abnormally prolonged seizures [4]. The consequences of this condition include increased mortality, neuronal injury, the alteration or disruption of neuronal networks, and increased morbidity [4]. Further consequences, which depend on the type and duration of seizures, include behavioral changes and cognitive impairment [4]. The main goal of treatment is to stop seizure activity to improve outcomes [4,5]. However, if SE is not controlled by initial anticonvulsant protocols, general anesthesia is required to control seizure activity. SE that is not controlled by the initial two anticonvulsant treatments is considered to be refractory and requires treatment with general anesthesia [4,5]. While the majority of refractory SE is controlled in this manner, some cases are refractory to treatment and continue to occur either with anesthesia or recur immediately after withdrawing general anesthesia. SE that progresses to this state is termed super-refractory status epilepticus (SRSE). SRSE is defined as SE that persists despite 24-hour treatment with intravenous anesthetic agents and recurs when weaning patients off these agents [4]. Although this condition is uncommon, it is an important clinical problem that requires innovative treatment approaches. Thus, it is important to understand its prognosis and recent advances in managing SRSE. Currently there are no clear protocols to treat SRSE, and this is an important area for future research initiatives. However, considerable research over the past several years has led to improved treatment protocols and several guidelines have been developed to treat and assist in managing this serious neurological emergency [3].

**Figure 1.**
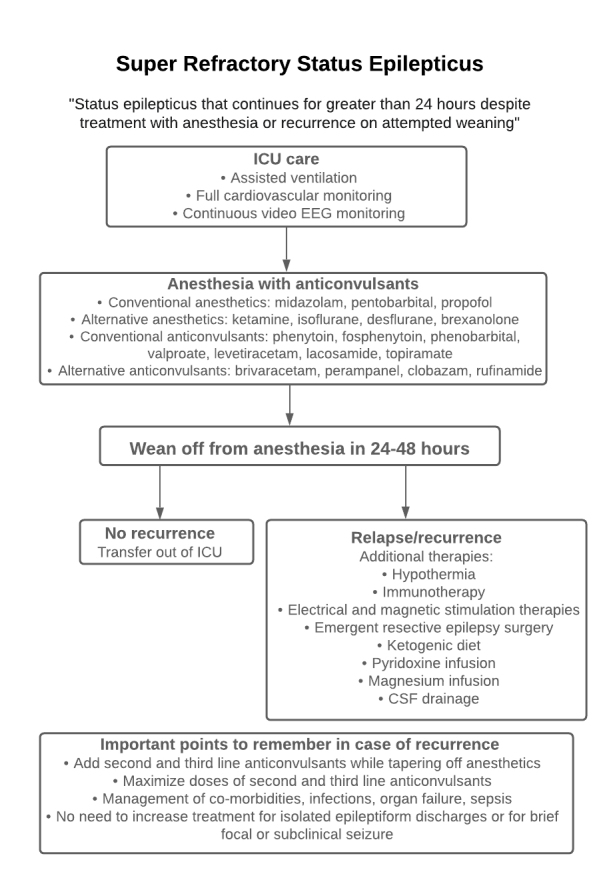
Management of SRSE.

## Therapeutic Approaches to SRSE

SRSE is treated in intensive care units (ICUs) equipped with assisted ventilation, full cardiovascular monitoring, and continuous video electroencephalography (EEG) monitoring. Anesthetic agents and anticonvulsants are used initially in its treatment. Other alternative options commonly used in refractory cases include resective surgery, neuromodulation devices, the ketogenic diet, pyridoxine infusion, cerebrospinal fluid drainage, and magnesium infusion (Fig. 1).

**Table 1:** Commonly used Anesthetics in SRSE.

Drug	Mechanism of action	Loading dose; Maintenance infusion rate	Adverse effects/Side effects	Caution	Ref.
**Midazolam**	Binds to GABA receptor-chloride complex in CNS, increases frequency of chloride channel opening, acts as muscle relaxant - rapid response	Loading dose: 0.2-0.4 mg/kg IV every 5 min until seizures are controlled with max dose 2 mg/kg Maintenance: 0.1-2.0 mg/kg/h Pediatrics: 0.15 mg/kg, additional doses of 0.1-0.3 mg/kg	Respiratory depression, hypotension, anterograde amnesia, drowsiness, ataxia	Obese patients, patients with renal insufficiencies, long-term use associated with lasting memory deficits	[6, 7, 8, 11, 13]
**Pentobarbital**	Strong anticonvulsant properties, similar to Midazolam but also causes inhibition of glutamate and intensifies depressant effects of GABA - longer duration of action	Loading dose: 5 mg/kg IV up to 50 mg/min every 5 min until seizures are controlled with max dose 15 mg/kg Maintenance: 0.5-5 mg/kg/h	Hypotension (patients require pressors), adynamic ileus, bowel ischemia, acidosis	Loss of brainstem reflexes and isoelectric pattern on EEG with high doses, distributes rapidly	[15, 16, 18, 19, 20]
**Propofol**	Modulates GABA receptor similar to midazolam and pentobarbital, greater ease of control, no serious drug-drug interactions	Loading dose: 3-5 mg/kg IV every 5 min until seizures are controlled with max dose 10 mg/kg Maintenance: 5-10 mg/kg/h	Hypotension, respiratory depression, injection site reactions, risk of PRIS	Recommended to limit use to no more than 3 days at a dose not to exceed 5 mg/kg/h, monitoring of lab values with prolonged use	[5, 23, 25, 27]

## Pharmacological Management of SRSE

Pharmacological management of SRSE remains the cornerstone of its treatment, and the timing of the initiated treatment is directly related to the severity of its prognosis. The more commonly used anesthetic agents and anticonvulsants are summarized in Table 1 and Table 2.

### 1. Anesthetic Agents

Anesthetic agents are the first choice of method for the treatment of SRSE. It is important to rapidly initiate general anesthesia following diagnosis as SRSE duration is directly correlated with a higher mortality and morbidity [1,2]. Familiarity with the use of these agents should assure the rapid termination of most SRSE cases. Midazolam, pentobarbital and propofol are the three most commonly used anesthetic agents at the bedside, and each have pros and cons for use in specific clinical circumstances.

#### Conventional Anesthetic Agents

##### Midazolam

Midazolam, along with pentobarbital and propofol, is one of the three conventional drugs used when first and second-line agents are unsuccessful [6]. Midazolam works by converting to its active metabolite, alpha-1 hydroxy midazolam via cytochrome P450 enzymes and glucuronide conjugation [7]. Similar to other benzodiazepines, midazolam exerts its effects by binding to the gamma-aminobutyric acid (GABA) receptor-chloride ionophore complex in the central nervous system (CNS) [7]. This allows for membrane hyperpolarization, increases the frequency of the chloride channel opening, and thereby increases the inhibitory effect of GABA [7,8]. This effect acts on glycine receptors and is a muscle relaxant and has predictable anxiolytic, amnestic, hypnotic, anticonvulsant, and sedative properties [7,8]. In the treatment of status epilepticus, it can be administered by intravenous bolus, continuous intravenous (C-IV) infusions, intramuscular injection, buccally, or nasally [8]. Due to its water-soluble nature, midazolam has a relatively short half-life compared to other benzodiazepines. For children, it has a half-life of 0.79-2.83 hours [9] and 1.36-4 hours in adults [10], making it a good choice for faster-acting pharmacokinetic properties and a reduced risk of toxicity. Due to its rapid response, midazolam is associated with tachyphylaxis and this renders it less effective with successive doses [9]. If used continuously, larger doses are needed to maintain its therapeutic effect. It has also been noted that caution should be taken in obese patients and those with renal insufficiencies as they are at risk for toxic accumulation [6].

**Table 2:** Commonly used Antiepileptics in SRSE.

Drug	Mechanism of action	Loading dose; Maintenance infusion rate	Adverse effects/Side effects	Caution	Ref.
**Phenobarbital**	Binds to and activates postsynaptic GABA_A_ receptors and decreases neuron excitability and reduces spread of seizure activity	Loading dose: 10 mg/kg up to 20 mg/kg, given at a rate of 100 mg/min up to 700 mg in 7 minutes	Depressive effect, severe sedation, hypotension, respiratory depression, cardiac arrhythmias, decreased GI motility, immunosuppression	Respiratory depression amplified when co-administered with benzodiazepines, high concentrations may result in reduced brainstem reflexes	[5, 27, 78,79]
**Phenytoin**	Obstructs positive feedback loop of Na channels to prevent further seizure spread, dependent on P450 enzyme system for metabolism	Primarily administered intravenously at a max rate of 50 mg/min	Neurotoxicity, cardiovascular toxicity, nystagmus, ataxia, lethargy, tremor, coma, seizures, bradycardia, hypotension, asystole	Pregnancy (FHS), poor water solubility inhibits complete absorption, nonlinear elimination, Purple glove syndrome, not suitable over other anticonvulsants	[55,56,57, 58,59,60]
**Fosphenytoin**	Similar to that of phenytoin with improved water solubility, allows for compatibility with IV solutions, both administered and eliminated rapidly	Max loading dose: 50 mg/min Pediatrics: 1-2 mg/kg/min or 20 mg/kg	Arrhythmia, hypotension, paresthesias, nystagmus, ataxia	For patients with renal/hepatic disease, a reduction of infusion rate by 25-50% is recommended; caution when other protein bound drugs are co-administered	[67,68,72-75]
**Levetiracetam**	Metabolic pathway is an enzymatic hydrolysis of the acetamide group, low protein binding	Loading dose: 1000-3000 mg over 15 min Maintenance: 2000-3000 mg/day	Somnolence, dizziness	Strength of recommendation is low, but may be better if used earlier	[95,101,102]
**Valproate**	Blocks Na channels and enhances GABA-mediated inhibition	Loading dose: 15-45 mg/kg as a bolus (6-10 mg/kg/min) Maintenance: 1-3 mg/kg/h	Dizziness, thrombocytopenia, mild hypotension	Acute encephalopathy and hyperammonemia	[ 83,84,85]
**Lacosamide**	Selectively enhances Na channels by slow inactivation and inhibits neuronal firing, high oral bioavailability, fast absorption	Loading dose: 200-300 mg administered over 15 min Maintenance: 200 mg every 12 hours	Headache, dizziness, diplopia, back pain, somnolence	Rate of adverse effects correlates to higher doses compared to shorter infusion times	[105,106,109]

The loading dose of midazolam in the treatment of refractory status epilepticus is 0.2-0.4 mg/kg IV every 5 minutes until seizures are controlled with a maximum dose of 2 mg/kg [6]. Its maintenance infusion rate is 0.1-2.0 mg/kg/h [6]. Adverse effects include respiratory depression, in which patients often require intubation and mechanical ventilation [10], and hypotension, in which 30-50% of patients will require pressors [11]. For the treatment of pediatric SE, a recent study involving 34 Japanese children was conducted in which an initial bolus of 0.15 mg/kg midazolam was given, with additional doses of 0.1-0.3 mg/kg to a cumulative dose of 0.6 mg/kg [12]. For high-risk patients, a continuous infusion at 0.1 mg/kg/h, with a maximum of 0.4 mg/kg/h, was administered [12]. This dosing regimen achieved a good cessation rate of 88%, suggesting that midazolam is suitable for pediatric patients as a first-line treatment [12].

In the elderly, midazolam is known to cause anterograde amnesia, drowsiness, and ataxia [7]. According to a study comparing 100 patients with similar baseline characteristics treated with high-dose (0.4 mg/kg/h) and medium-dose (0.2 mg/kg/h) continuous midazolam infusion, withdrawal seizures within two days of discontinuation were less frequent in the high-dose group [13]. High-dose C-IV treatment of SRSE can be performed safely and is associated with fewer withdrawal seizures and better efficacy for lasting seizure control after discontinuation [13].

As with all aggressive anesthetic treatments, it is recommended that midazolam is used to suppress seizures for 12 to 24 hours, followed by a gradual taper off the medication, continuous monitoring of blood levels and its metabolites, and EEG monitoring to identify changes in brain function which may not be evident by neurological examination alone [8]. Long-term use of midazolam is cautioned against as it is associated with lasting memory deficits [8].

##### Pentobarbital

Thiopental and its metabolite pentobarbital have strong anticonvulsant properties and are widely used in the treatment of SRSE. Pentobarbital is one of the oldest anticonvulsants used to induce pharmacologically induced anesthesia. It works in the CNS much like other benzodiazepines. It binds to GABA_A_ subtype receptors which induces a change in the chloride transport receptor leading to an increase in the duration of opening of the chloride channels which potentiates the effects of GABA. GABA causes CNS depression and since the channels remain open for the longer duration, the depressant effects of GABA are intensified [14]. Pentobarbital also causes inhibition of glutamate, which is responsible for nerve depolarization in the voltage-activated calcium currents which has a synergistic effect of causing depression. It follows the first pass hepatic metabolism and has many drug interactions [14,15,16]. Pentobarbital affects anticoagulants, predominantly warfarin, and other drugs such as levothyroxine, corticosteroids, doxycycline, phenytoin, valproic acid, alcohol, monoamine oxidase inhibitors, estradiol, estrone, and progesterone [14]. GABA subtype receptors also lower the body temperature and have neuroprotective effects. Loss of brainstem reflexes and an isoelectric pattern are seen on EEGs with high doses of pentobarbital [17].

Pentobarbital’s loading dose in SRSE is 5 mg/kg IV up to 50 mg/min every 5 minutes until seizures are controlled, or a maximum loading dose of 15 mg/kg. The maintenance infusion rate is 0.5-5 mg/kg/h [18]. It is effective in cessation of seizure activity in most cases [18,19]. The retrospective study by Pugin and colleagues treated 31 patients with SRSE with pentobarbital, and 90% of patients achieved seizure control. Seizures recurred upon weaning of the drug in 48% of patients, however the outcome in this cohort of patients remained poor due to underlying etiologies [19].

Pentobarbital is not a first-line treatment due to its prolonged duration of action, longer recovery time, and need for a longer duration of mechanical ventilation due to its rapid redistribution and zero order kinetics [20].

Patients treated for SRSE with pentobarbital require pressors as hypotension occurs in 29-77% of cases [19,20]. Adynamic ileus is also seen in up to 10% of patients, which can result in bowel ischemia or even bowel perforation [21]. Propylene glycol toxicity can be seen in 1% of patients and manifests as acidosis [19].

##### Propofol

Intravenous propofol is the last of the three most common anesthetic agents used in the management of SRSE [22]. Propofol is a potent depressant of the CNS. Its mechanism is poorly understood, but it likely exerts its effects by modulating the GABA_A_ receptor [4]. It is believed to decrease the dissociation of GABA from its receptor, which increases the duration of the chloride channel opening and causes an inhibitory effect on neurons [23]. Propofol allows for a greater ease of control of anesthesia compared to midazolam and pentobarbital due to its rapid onset of action and recovery despite prolonged infusion [4]. In addition to its advantageous pharmacokinetic properties, another benefit of propofol is that it has no serious drug-drug interactions [24]. There is also a lower occurrence and severity of hypotension and cardiorespiratory depression compared to midazolam and pentobarbital [4,24].

The propofol loading dose in the management of SRSE is 3-5 mg/kg IV every 5 minutes until seizures are controlled, with a maximum dose of 10 mg/kg [5, 24]. The maintenance infusion rate is 5-10 mg/kg/h [24,25]. Adverse effects include hypotension, with 22-55% of patients requiring the use of pressors, as well as respiratory depression and injection site reactions [4]. The main hazard during prolonged infusion is the risk for propofol infusion syndrome (PRIS) [4]. This is a rare complication, but it is of particular concern in children and in patients who are receiving concurrent steroids or catecholamine therapy [25]. PRIS is thought to result due to impaired mitochondrial activity [5]. It leads to severe metabolic acidosis, hyperkalemia, hyperlipidemia, rhabdomyolysis, cardiac dysfunction, and renal failure [5]. Due to its high morbidity and mortality rate, it is recommended to limit the use of propofol to no more than 3 days at a dose that does not exceed 5 mg/kg/h [5,26]. During prolonged use, laboratory values including serum levels of creatine kinase, lactate, and lipids should be monitored [22]. If PRIS is suspected, infusion should be stopped as the cascade of events that follows often results in fatal outcomes [27,28,29].

In a meta-analysis examining the results of different interventions in SRSE, it was reported that the rate of initial control on propofol was 68% [24]. 6% of patients on propofol required a change in therapy due to side effects, and breakthrough seizures (recurring seizures after a period of control) occurred at a rate of 1.3% [24]. Another meta-analysis found that in seven studies examining patients with refractory SE, propofol had a shorter average control time and a reduced average tracheal intubation placement time in comparison to barbiturates [30].

#### Alternative Anesthetic Agents

It is rare that SRSE is not controlled with midazolam, pentobarbital, and/or propofol. However, there are resistant cases and situations in which the patient is unable to tolerate these general anesthetics. The following alternative anesthetic agents have been used in these rare situations.

##### Ketamine

Ketamine is an anesthetic agent that is a potent N-methyl-D-aspartate (NMDA) receptor antagonist and has evidence of efficacy in advanced stages of SRSE. It is a good alternative to propofol but is reserved as a second-line drug due to limited clinical evidence and its potential neurotoxic effects. Its mechanism of action involves non-competitive blocking of NMDA receptor channels which confers different properties from those of other anesthetic agents. It induces dissociative anesthesia by activating the limbic system and disconnecting the thalamocortical pathways. Due to its low risk of cardiac depression and hypotension, it is the agent of choice in emergencies which include cardiocirculatory instability [31,32,33]. Ketamine also has a neuroprotective effect through the inhibition of NMDA receptor-induced glutamate excitotoxicity associated with increased intracellular Ca^2+^ influx and consequential cell death [33].

Gaspard and his colleagues investigated the use of ketamine in seven patients critically ill with SRSE and found that the drug produced electroencephalographic control of the crisis in 50% of cases without causing hemodynamic instability [34]. Shet and Gidal found that the use of ketamine in refractory cases was able to keep seizures under control [31]. Furthermore, Gaspard et al. showed in a retrospective study that the use of ketamine in refractory status epilepticus resulted in 57% (34/60) of cases in which seizures were resolved, 32% (19/60) of cases in which seizures were terminated, and approximately 13% (8/60) of cases in which seizures were controlled with the administration of intravenous ketamine [34]. The loading dose of ketamine is 1-3 mg/kg and the maintenance dose is 5-7.5 mg/kg/h [35].

The only absolute contraindication to the use of ketamine is the induction of tachycardia or a blood pressure crisis in hypertensive or non-stabilized coronary patients due to norepinephrine release from synaptic terminals. If the patient has depleted norepinephrine levels, the observed effect is myocardial depression with hypotension. However, these phenomena are less frequent with subanesthetic doses or continuous IV infusion and are generally controlled with the administration of benzodiazepines or haloperidol [36]. Paradoxically, ketamine has been related to both neuroprotection and neurological damage. Initially, it was thought that the use of ketamine in patients with cerebral damage should be avoided as it causes increased intracranial pressure (ICP). Studies have since shown that in addition to causing an increase in ICP, ketamine also increases cerebral oxygen consumption and cerebral flow. Today, the role of ketamine in the treatment of patients with neurological injury has been reconsidered. The negative effects in cerebral hemodynamics do not occur if ventilation is controlled and co-administered with GABAergic medications. Neurotoxicity from ketamine can be due to excessive receptor antagonism which makes pyramidal neurons of posterior cingulate, retrosplenial cortices, and Purkinje cells of the cerebellum prone to cell death [37]. In the younger population, ketamine can induce neuronal cell death in immature brains and cause altered neurogenesis in developing brains [38]. In patients with SRSE, it was seen that an earlier initiation and longer infusion of ketamine can improve its effect and the clinical outcomes [39]. Lastly, ketamine is a racemic mixture of enantiomers (*S*)- and (*R*)-Ketamine. (*S*)-Ketamine’s different pharmacodynamics makes it a more potent analgesic than the later enantiomer; however, treatment with (*S*)-Ketamine for patients with SRSE did not result in a higher efficacy than the racemic mixture of ketamine [40].

##### Isoflurane

Isoflurane, an inhalation anesthetic, has been shown to have promising effects in treating SRSE. Although its mechanism is still not fully understood, its effects are likely due to the potentiation of inhibitory postsynaptic GABA_A_ receptor-mediated currents [41]. Isoflurane, being resistant to biotransformation, has reduced potential for toxic effects on various organs. Furthermore, its rapid onset of action makes isoflurane an ideal drug for the treatment of SRSE [42].

Epileptic discharges are seen to help with continuous electroencephalographic burst suppression within minutes of starting therapy with isoflurane [43]. The typical dose is the minimal alveolar concentration of 1.0, and it can be decreased to 0.7 when accompanied with other drugs without causing hemodynamic instability [44]. In a study looking at the therapeutic effects of isoflurane, complications included hypotension, atelectasis, infections, paralytic ileus, and deep venous thrombosis for all or some of the patients. However, there was no development of renal or hepatic dysfunction [42]. Hypotension can be corrected with normal saline infusion and low dose vasopressors [44].

##### Desflurane

Inhaled desflurane is commonly used in the induction and maintenance of general anesthesia in adults [45]. It gained popularity due to its low blood solubility, allowing for rapid induction, and low risk of organ toxicity, making it safe for long-term administration [45,46]. The mechanism is not well understood, but desflurane is believed to inhibit excitatory channels (NMDA receptors) and potentiate inhibitory channels (GABA_A_ receptors) [46,47].

Inhaled anesthetics such as desflurane have been used in patients who do not respond to the conventional IV anesthetics. However, little is known about the effects of desflurane on SRSE. The largest case series examining the use of desflurane in RSE demonstrated effective electroencephalographic burst suppression in seven patients [43]. These patients received desflurane anesthesia for an average of 11 days, with a maximal end-tidal concentration ranging from 1.2-5.0%. Four patients showed good outcomes. The other three died from different causes - one from acute hemorrhagic leukoencephalitis, one from bowel infarction, and one from toxic encephalopathy [43]. Complications that affected all seven patients included hypotension and atelectasis. Five of the patients developed infections, three developed paralytic ileus, and two developed deep venous thrombosis [43].

Due to the high risk for complications and limited evidence of its efficacy, desflurane is generally not recommended in the treatment of SRSE [4].

##### Brexanolone

Brexanolone gained prominence as the first drug to be specifically approved by the FDA in March 2019 for intravenous use in the treatment of postpartum depression in adult women [48]. It is a solution of aqueous allopregnanolone in sulfobutylether-beta-cyclodextrin (SBECD), which may then be diluted and administered intravenously and has been used as adjunctive therapy in SRSE [49]. The exact mechanism of brexanolone is not clear, however it is known that allopregnanolone is an endogenous neuroactive steroid which plays a role in positive allosteric modulation on GABA_A_ receptors containing the δ subunit in place of the γ subunit and is responsible for modulating neuronal excitability [50,51]. This mechanism is unique compared to other benzodiazepines due to its ability to mediate tonic inhibition rather than phasic inhibition, and these δ subunit-containing GABA_A_ receptors have been shown to be extremely sensitive to allopregnanolone, but comparatively insensitive to other benzodiazepines [52]. In a study investigating the combination efficacy of neurosteroids, the combination of brexanolone and tiagabine (TG) in fixed ratios of 1:1, 3:1, and 1:3 demonstrated robust protective synergistic interactions [53]. These results are likely due to the additive antiseizure activity at both synaptic and extrasynaptic GABA_A_ receptors [53].

As a newer drug with few documented cases and use in clinical trials, available information regarding its use in SRSE is limited. However, in a 5-day infusion open-label cohort in which brexanolone was added to the standard-of-care for SRSE, patients showed good tolerance accompanied by a high rate of successful weaning from third-line anaesthetic agents [49]. There was no evidence of a significant effect on vital signs of patients due to brexanolone [49]. Due to these results, it has been suggested that brexanolone is used as a possible adjunctive therapy for SRSE patients who require pharmacological coma in order to control their seizures. Furthermore, this preliminary study demonstrated that brexanolone is well tolerated in a heterogeneous population [49].

### 2. Antiepileptic Agents

Antiepileptic agents are used in the initial treatment of SE. The agents reviewed in this section represent the most commonly used treatments for the onset of SE. Failure to control SE with two main antiepileptic agents identifies RSE and the need for the use of general anesthetic treatment. The initial antiepileptic agents should be maintained during general anesthesia and during the treatment of SRSE. When weaning off the general anesthetic agents, it is important to establish that therapeutic levels of antiepileptic agents are present and that they are available should the SE return.

The choice of antiepileptic agents is usually based on the choice of the treating physician. In stage 1 (early) SE, the first line therapy is benzodiazepines. Stage 2 SE occurs when there are continued seizures despite benzodiazepine therapy, and treatment in this stage is with IV anti-seizure drugs, including phenobarbital [4]. Other anti-seizure drugs include phenytoin, fosphenytoin, valproate, levetiracetam, and lacosamide, but there is no strong evidence of one option being more effective than the other [54]. Randomized clinical trials to study the efficacy of levetiracetam, fosphenytoin, and valproate for established SE by age group showed that children, adults, and older adults respond similarly to all three drugs with treatment success in approximately fifty percent of patients. Any of the three drugs can be considered as a first-choice, second-line drug for benzodiazepine-refractory SE [54].

#### Phenytoin

Phenytoin is a first-generation anticonvulsant drug that was approved in 1939 for the treatment of epilepsy and is one of the most well-studied anticonvulsants [55,56]. It is administered orally or parenterally. When administered intravenously, it is delivered into a large central or peripheral vein at a maximum rate of 50 mg/minute [56]. By obstructing the positive feedback loop of sodium channels, which are responsible for sustaining high-frequency action potentials, phenytoin prevents the further seizure spread [56].

Phenytoin has a half-life of 22 hours, is absorbed entirely, and reaches a peak concentration at 1.5 to 3 hours [56]. However, its complete absorption may exceed two weeks due its poor water solubility which subsequently reduces its motility within the gastrointestinal tract [55,57]. Its prolonged presence is also attributed to its nonlinear elimination in which it follows first-order kinetics at plasma concentrations below 10 mg/L and changes to zero-order kinetics following saturation in the body [56]. Due to its dependency on the hepatic P450 enzyme system to metabolize phenytoin to its pharmacologically active form, drugs that alter the function by either inhibiting or inducing these enzymes must be monitored based on resulting phenytoin levels [55, 57,58].

Adverse effects include neurotoxicity, associated with oral administration, and cardiovascular toxicity, associated with parenteral administration [59, 60]. Neurotoxic effects are concentration-dependent and range from occasional mild nystagmus, ataxia, lethargy, tremor, to coma and seizures at high concentrations [59]. Cardiovascular toxicity effects due to rapid infusion can lead to bradycardia, hypotension, and asystole [60,61]. Due to its formulation, it is possible for crystallization of phenytoin to occur within the blood [57]. While rare, this may lead to purple glove syndrome, in which there is extensive skin necrosis and limb ischemia [57]. The only absolute contraindication is pregnancy [57]. This is due to the development of fetal hydantoin syndrome (FHS) in pregnant women who were administered phenytoin [57].

Due to the nature of phenytoin, its narrow mechanisms of action, complex pharmacokinetics, drug-drug interactions, unique adverse effects, and formulation utilizing propylene glycol as its vehicle, which itself may produce serious cardiovascular complications, phenytoin requires proper management to monitor patients for neurological or cardiac toxicity and to limit the rate of its absorption. While phenytoin has been well-used for more than half a century, the general consensus is that there is insufficient evidence-based data to support its efficacy over other anticonvulsants [62]. For example, it has been found that when used alone, phenytoin is inferior to lorazepam, phenobarbital, or diazepam, and is as effective or more so as levetiracetam and valproate [63-66]. As such, phenytoin is associated with many clinically significant disadvantages.

#### Fosphenytoin

Fosphenytoin is a water-soluble disodium phosphate ester of phenytoin that was approved by the FDA in 1996 for use in epilepsy [67]. It is similarly a long-acting anticonvulsant drug used to prevent recurrent convulsions, though it is also used as a short-term solution in acute scenarios [68,69]. Its mechanism of action is similar to that of phenytoin as it was developed to mimic its effects, but with improved water solubility [70]. It works by rapidly converting to the active form of phenytoin by plasma and tissue esterases after its intravenous administration [67,71]. It was developed as an improved alternative to phenytoin as its formulation allows its compatibility with common IV solutions and can be safely administered intramuscularly [68,72]. Since being developed, fosphenytoin’s pharmacokinetics have demonstrated a similar efficacy to that of phenytoin, but has been associated with fewer adverse effects, more convenient intravenous administration, and available for intramuscular injection [67,68,72-74]. While fosphenytoin is a more costly option, the complications due to using its predecessor may also be costly.

Fosphenytoin is administered either intravenously or intramuscularly, but the latter is not the preferred route for SRSE or for pediatric patients [75]. Following intravenous administration, a therapeutic effect is observed immediately, and following intramuscular administration, a therapeutic effect is observed within 30 minutes [67]. Its maximum plasma concentration is achieved within 90-190 minutes following intramuscular administration [70]. The plasma concentration profiles for both children and adults match closely and are comparable in terms of doses and infusion rates [67]. Unlike its predecessor, fosphenytoin is both administered rapidly and eliminated rapidly with a half-life of ranging from 7-15 minutes [67, 68]. Similar to phenytoin, the maximum recommended loading dose of fosphenytoin is 50 mg/min [67]. For pediatric patients, the recommended administration rate is 1-2 mg/kg/minute [75]; a loading dose of 20 mg/kg has shown efficacy and a good safety profile in a majority of children with SE [76]. In emergency situations, the rapid administration of fosphenytoin is a recommended option [67].

Adverse reactions include arrhythmia, hypotension, paresthesias, nystagmus, and ataxia [70]. For patients who suffer from renal or hepatic disease, hypoalbuminemia, or are elderly, a reduction in the infusion rate by 25-50% and close monitoring is recommended due to their decreased ability to bind fosphenytoin [67]. Similar to phenytoin, caution must be taken when other highly protein bound drugs are co-administered.

#### Phenobarbital

Phenobarbital is a barbiturate anticonvulsant that also functions as a sedative hypnotic [77]. It was introduced as a sedative at the start of the 20^th^ century, and its antiepileptic effects were discovered soon after its introduction to the market [27]. By 1919, it was recommended for the treatment of SE [27]. Phenobarbital works by binding to and activating postsynaptic GABA_A_ receptors [27]. It extends the amount of time that chloride channels are open, therefore hyperpolarizing the cell membrane and decreasing neuron excitability [5,27]. This increases the action potential threshold and reduces the spread of seizure activity [6]. The half-life of phenobarbital is around 72-144 hours in adults [5]. Phenobarbital is water-soluble and is metabolized by the liver and excreted mainly by the kidneys [5,77].

Phenobarbital can be administered orally, intramuscularly, or intravenously [78]. The intravenous route is preferred for emergency situations, including SE [78]. For adults, the recommended loading dose is 10 mg/kg up to 20 mg/kg, given at a rate of 100 mg/min up to 700 mg in 7 minutes [79].

The duration and dose of phenobarbital is limited by its central depressive effect [5]. Adverse reactions include severe sedation, hypotension, respiratory depression, cardiac arrhythmias, decreased GI motility, and immunosuppression [5,79]. Respiratory depression is amplified when phenobarbital is co-administered with benzodiazepines [79]. High serum concentrations may result in diminished brainstem reflexes [5].

A meta-analysis which evaluated the efficacy of different antiepileptic drugs in 798 cases of convulsive benzodiazepine-resistant SE found that phenobarbital had a 73.6% success rate [80]. Mega-dose phenobarbital has been shown to be an option in treating adults with SRSE that is resistant to other treatments, with a successful outcome in 5 out of 10 patients [81]. Additionally, evidence has shown that high-dose phenobarbital therapy can mediate safe withdrawal of pentobarbital anesthesia, making it a treatment option in cases of unsuccessful pentobarbital-induced coma in SRSE [82].

#### Valproate

Valproic acid (VPA) is used in the treatment of SRSE with a dosage of up to 40 mg/kg/min, which is tolerated well in most patients. The effective dose ranges from 15-45 mg/kg as a bolus (6-10 mg/kg/min) followed by 1-3 mg/kg/h infusion [83]. Intravenous VPA has good tolerability in terms of cardiovascular and respiratory status in patients with SE. Adverse side-effects are seen in less than 10% of patients, and the most common side-effects include dizziness, thrombocytopenia, and mild hypotension [84]. The most concerning adverse effects include acute encephalopathy and hyperammonemia [85].

There has been data which show that IV VPA has been effective in the treatment of SE. This data is based on six randomized controlled trials (RCTs) with SE in which sodium valproate efficacy was studied and compared with other intravenous anticonvulsants including phenytoin, diazepam, and phenobarbital [86-90]. Furthermore, Trinika et al. summarized 30 studies of IV VPA in a total of 860 patients, showing an overall response rate of 71% in aborting SE [84]. Overall, valproate is a good choice as a second-line anticonvulsant.

#### Levetiracetam

Levetiracetam (LEV) is a well-established second-generation anticonvulsant that is not associated with significant drug interactions and contains a good pharmacokinetic profile, making it particularly efficacious against SE [91-94]. Zhan Miao-Yi et al. conducted a systematic review and meta-analysis of efficacy, safety, pharmacokinetics and economics for intravenous LEV which showed it has similar efficacy as lorazepam, phenytoin, and valproate for SE. LEV has a decreased need for ventilator assistance and significantly less risk of hypotension which further supports that it is a favorable choice for second-line treatment [95]. IV LEV is also found to be efficacious in the management of acute seizure management and SE in children, neonates and preterms in terms of tolerability and efficacy [96-101]. The initial IV loading dose is 1000 to 3000 mg [102]. Fewer side-effects are seen as compared to other anticonvulsants, with sedation and thrombocytopenia seen in rare cases [102].

#### Lacosamide

Lacosamide is a novel antiepileptic drug that was approved in 2008 by the FDA and EMA [103]. It is used as an adjunctive treatment for partial-onset seizures [104]. Lacosamide is a functionalized amino acid with a unique mechanism of action [105]. It selectively enhances voltage-dependent sodium channel slow inactivation, thereby stabilizing hyperexcitable neuronal membranes and inhibiting neuronal firing [106]. Additionally, it modulates the collapsing response mediator protein 2 (CRMP-2), although the role of this binding is not well understood [107]. Lacosamide has a high-water solubility and is available as an oral formulation and as an IV solution [105]. It has a favorable pharmacokinetic profile, including fast absorption, linear pharmacokinetics, and high oral bioavailability [106]. It also has low drug-drug interaction potential [108]. Adverse side effects are rare and typically mild, with the most common complaints including headache, dizziness, diplopia, back pain, somnolence, and injection-site pain [105].

Intravenous lacosamide is bioequivalent with oral formulations [106]. It is typically used as second- or third-line therapy in RSE after the failure of benzodiazepines, levetiracetam, and phenytoin [104]. A trial investigating safety and tolerability reported an optimal tolerance at a loading dose of 200-300 mg administered over 15 minutes [105]. Another trial reported a typical regimen consisting of an initial loading dose of 200 mg followed by 200 mg every 12 hours [109]. The rate of adverse effects correlates with higher doses of lacosamide rather than shorter infusion times [105].

In a retrospective analysis of 39 patients with SE, it was found that lacosamide had a 60% success rate when administered early during the treatment course and a 20% success rate in more refractory cases [103]. There were no adverse events other than one case of an allergic rash [103]. A systematic review evaluated studies reporting on lacosamide use in SE and found that it had an overall efficacy of 57% in a total of 522 cases [106]. The efficacy with later positioning (RSE) was found to be 20% [106]. Current data indicates that lacosamide is a useful option in the treatment of non-refractory and refractory SE, with an efficacy similar to other antiepileptic drugs in more refractory cases [110].

#### Topiramate

Topiramate is an effective anticonvulsant with a low incidence of adverse effects. Its kinetics are linear, with a half-life of 21 h, low protein binding, no enzyme autoinduction, and no active metabolites [111]. It is eliminated via the kidney, mainly as an unmodified drug. It has several mechanisms of action: sodium and calcium channel blockade, GABAergic action, glutamatergic antagonism, and inhibition of the enzyme carbonic anhydrase [111]. Suspension of topiramate is typically administered via nasogastric tube. It has limited use in the context of SRSE, and few cases in RSE.

Towne et al. present a series of 6 adult patients in RSE who responded to oral topiramate in doses that ranged from 300 to 1,600 mg/day between 6 h and 10 days after starting the drug. The patient who took the longest to respond also had the longest RSE (38 days) and the highest dose (1,600 mg/day). The only complication reported was drowsiness [112]. In addition, Bensalem et al. described three patients who responded to topiramate in two days with a dose of 1 g/day for 2 to 5 days, and was gradually reduced in subsequent days without complication [113]. This therapy has also been used in children with similar results [114].

#### Newer Antiepileptic Agents

##### Brivaracetam

Brivaracetam is one of the newest antiepileptic drugs and is an analog of levetiracetam. It is a high-affinity synaptic vesicle glycoprotein 2A ligand and regulates neurotransmitter release by selectively binding to the synaptic vesicle protein 2A (SV2A) transporter for galactose [115,116].

In a retrospective multicenter registry of SE cases at seven hospitals in Spain, it was observed that brivaracetam delivered at median loading doses of 100 mg and weight-adjusted at 1.8 mg/kg was effective within 6 hours in 54% of patients with an overall median response time of 22 hours [115]. If delivered earlier and at higher doses (300 mg vs 100 mg and weight-adjusted at 3.85 mg vs 1.42 mg, with the best cutoff point at 1.82 mg/kg), it had a tendency to be more effective [115].

The rapid pharmacokinetics of brivaracetam along with its low potential for clinically relevant drug-drug interactions and adverse consequences make it a strong candidate for use in emergency situations. It has shown promise in animal models, with potent anti-seizure and anti-myoclonic activity and anticonvulsant effects [116]. Due to its novelty, there is currently a lack of clinical trials assessing brivaracetam in SRSE in humans in a standardized manner. With the small number of published cases, we are unable to draw any conclusive conclusions as any negative or positive outcomes may have resulted from synergistic actions of other treatments [117].

##### Perampanel

Perampanel is used for the adjunctive treatment of epilepsy up to 12 mg/day and acts as an orally active non-competitive α-amino-3-hydroxy-5-methyl-4-isoxazole-propionic acid (AMPA) receptor antagonist to reduce glutamate-mediated postsynaptic excitation [118-120].

In a retrospective review of 52 patients administered perampanel as a last drug for SE in five European hospitals, it was found that the median latency from SE onset to perampanel initiation was 10 days. The median initial dose was 6 mg/day up to a maximum dose of 10 mg/day. The overall rate of seizure cessation attributed to perampanel was 36.5% [121]. Newey et al., reported results in four patients treated with high doses of perampanel for SRSE resulted in a reduction of seizure burden without affecting hemodynamics, hepatic, or renal functions [122]. Thus, while a newer treatment, there is good evidence that perampanel may be an effective treatment in patients who have failed multiple AEDs or have SE of varying etiologies.

##### Clobazam

Clobazam is an orally active benzodiazepine that has anticonvulsant properties. Clobazam increases the presynaptic inhibition of neurons, thereby limiting the spread of electrical activity, although they do not inhibit the discharge of the abnormality [123]. Clobazam is rapidly and extensively absorbed after oral administration. The typical dose ranges from ≤1.5 mg/kg/day to 1 mg/kg/day [124]. The most common adverse drug reactions include sedation, headache, nausea, aggression, irritability, ataxia, constipation, dry mouth, blurred vision, depression, insomnia, and amnesia [125].

Dr. Sivakumar and colleagues identified 17 patients with RSE who were treated with clobazam. Their treatment showed a favorable pharmacokinetic profile devoid of drug interactions. Seization of RSE within 24 hours of administration, without addition of other drugs, was seen in 13 patients. Clobazam was well tolerated in the treatment of RSE and appears to be an effective and promising drug option for SRSE [126].

##### Rufinamide

Rufinamide is a novel anticonvulsant that was approved by the FDA in 2008 as an adjunctive agent in the treatment of Lennox-Gastaut syndrome [127]. Lennox-Gastaut syndrome (LGS) is a rare form of epilepsy that begins in childhood and results in developmental delays and multiple seizure types [128]. In addition to LGS, rufinamide has also shown some effectiveness in treating other refractory epilepsy syndromes, although research in this area is limited [128].

There has been one case report in which rufinamide was used in the treatment of SRSE. In this report, the subject was a 24-year-old man with refractory tonic status epilepticus. Prior to the use of rufinamide, nine AEDs along with hypothermia were used in an attempt to terminate seizure activity. Rapidly titrated rufinamide, up to a total dose of 3 g/day, was effective in controlling the seizures and allowed for discontinuation of other agents [129]. This study suggests that rufinamide may be useful in the treatment of SRSE, although more research is needed.

## Additional Options for the Treatment of Super-refractory Status Epilepticus

### 1. Hypothermia

Therapeutic hypothermia has been successful as a measure of brain protection for those who suffer from cerebral hypoxia due to cardiorespiratory arrest, or with difficult-to-manage cerebral edema. It has found less success in patients with severe head trauma. The use of this therapy in SE is an extreme measure when antiepileptic drugs and anesthetics have failed [130]. In 1984, Orlowski et al. published their experience with three pediatric patients, who were subjected to barbiturate coma and moderate hypothermia (30-31 °C) for controlling SE [131]. All three patients were first treated with thiopental to achieve burst suppression. Hypothermia was then induced with surface cooling and patients were kept in a state of burst suppression for 48-72 hours, titrating the thiopental as necessary. After this period, thiopental was discontinued, and the patients were slowly rewarmed. Two of the three patients made a total or near total recovery without seizure recurrence, while the third later died and was diagnosed with Rassmussen’s Encephalitis at autopsy [131]. When using hypothermia, anticonvulsant levels should be monitored because it significantly reduces drug clearance by reducing the activity of the cytochrome P450 system, cardiac output, and glomerular filtration [132].

### 2. Steroids

There has been growing experimental evidence that systemic inflammation plays a role in epilepsy. Activation of signaling pathways including the toll-like receptor-interleukin 1 receptor signaling network is suggested to be a factor in epileptogenesis [4]. Additionally, induced SE in animal models has been found to increase transcript levels of inflammatory markers such as IL-1B, IL-6, and TNF-a [133]. These discoveries suggest that patients with SRSE may benefit from immunological therapy with high dose steroids as a second-line treatment. In addition to their immunological effects, steroids may benefit these patients by reversing the blood-brain barrier opening, which contributes to persistent seizure activity [4]. They may also have positive effects on cerebral edema and intracranial pressure [134]. Therapy with high-dose steroids is usually attempted with 1 g/day of intravenous prednisolone for 3 days, followed by 1 mg/kg/day for about a week and continued for a longer term if there is a response [25, 26]. Adverse effects of steroids may include gastrointestinal ulceration, hypertension, hyperglycemia, and fluid retention [134].

A recent review found that IV methylprednisolone pulse had “positive effects” in 17% of 63 treatments in patients with NORSE and 38% of 38 treatments in patients with febrile infection-related epilepsy syndrome [135].

### 3. Immunosuppressive Agents

Autoimmune refractory status epilepticus is a condition in which autoantibodies interfere with receptors and ion channels within the brain, resulting in encephalitis and recurrent seizures [136]. This rare form of SE is refractory to anticonvulsive treatment with immunomodulatory therapies as the mainstay of treatment [136]. It is appropriate to treat patients with this condition with immunosuppressive drugs, including corticosteroids and IV immunoglobulins. In patients that do not respond to these treatments, the use of second-line agents such as rituximab and cyclophosphamide may be indicated [137].

A recommended second-line therapy is 375 mg/m^2^ of rituximab every week for 4 weeks, and 750 mg/m^2^ of cyclophosphamide given with the first dose of rituximab and administered monthly [136,137].

In the case of a patient with RSE caused by anti-NMDA receptor encephalitis, combination therapy with cyclophosphamide and rituximab resulted in a marked improvement of her condition [138]. A retrospective review of patients with new onset RSE reported improvement upon intervention with intravenous cyclophosphamide, although partial seizures persisted [139]. Given the success of these immunosuppressive agents in the treatment of RSE, they show similar promise in the treatment of SRSE.

### 4. Electrical and Magnetic Stimulation Therapies

#### a. Electroconvulsive Therapy

Electroconvulsive therapy (ECT) has been widely applied in refractory psychiatric diseases, such as depression or schizophrenia, with medically documented safety and efficacy [140]. While its effects require further investigation, ECT has been recommended as a nonpharmacologic option of treatment for SRSE if other alternatives are unsuccessful. The exact mechanism of ECT is unclear, but it has been proposed that its benefits on seizure-based disorders are associated with the activation of endogenous GABAergic pathways, promotion of neurotrophic factors, and decrease in neural metabolism [141,142].

In a recent case series, ECT was applied to six patients with SRSE after the failure of antiepileptic treatment and pharmacologic coma. Electrodes were placed on bilateral frontotemporal regions and up to three stimuli were provided per ECT session at an initial stimulus of 500 mC up to a maximum of 1000 mC. The frequency of stimulation was 60 Hz, and the duration of the stimulus was 5 seconds. It was found that SRSE was resolved in all patients after several days of treatment without significant adverse effects [143]. These results were consistent with several other case reports in both children and adults, one of which found successful SRSE cessation even after six weeks of prolonged SE and exhausted anticonvulsant pharmacotherapeutic strategies [141,144-146].

The minimal use of ECT in the management of SRSE may be attributed to its lack of data and its limited availability in ICU settings. While ECT has been recommended as a last resort, some investigators have pointed out that earlier interventions and high energy charges with repetitive seizure simulations would yield more favorable outcomes as there would be less excitotoxic damage, lower downregulation of the inhibitory system of the brain, and less risk for systemic infection [141,147]. Much of the literature notes that patients who received this treatment had received various other AEDs and anesthetics previously. Therefore, the individual contribution of ECT is unclear. Despite the lack of controlled clinical trials, several case reports suggest that ECT is both safe and effective. Furthermore, due to its noninvasive nature and minimal side effects, ECT appears to be a reasonable option in the treatment of SRSE.

#### b. Transcranial Magnetic Stimulation

Transcranial magnetic stimulation (TMS) is used in SE as an alternative treatment when conventional treatments fail. It is a non-invasive technique in which pulsed intracranial electrical current is induced by electromagnetic induction to suppress cortical activity at low frequencies (≤ 1 Hz), and cortical excitability at high frequencies (≥ 5 Hz) [148]. It can be used to transiently disrupt the function of the targeted cortical region, map out functionally relevant brain regions, and assess cortical reactivity [149]. When applied repetitively, it is possible to modify cortical excitability and produce therapeutic effects. A majority of relevant medical literature consists of case reports and series, with available data suggesting that the risk from TMS in children is similar to that in adults [150].

In three cases of RSE treated with repetitive transcranial magnetic stimulation (rTMS) in the ICU, rTMS was found to decrease seizure frequency [148, 151]. In another notable case report, a 24-year-old young man was treated with rTMS therapy approximately 14 months after his initial generalized convulsion and five months after his seizure frequency increased [152]. He received 11 sessions over 10 days, each session consisting of three 10-minute trains of 1 Hz pulses. After the first few days of treatment, his number of electrographic seizures markedly declined and maintained at zero seizures per day [152]. Post-discharge, he experienced relatively few interictal discharges and underwent maintenance rTMS sessions identical to his inpatient course. Nine months following his initial round of rTMS found no apparent progression of the underlying epilepsy syndrome [152]. It has been noted that the anti-seizure effect observed may be due to the fact that the low-frequency rTMS protocol induced synaptic plasticity distinct from the pathways used in traditional AEDs [150,152].

While rTMS is well-tolerated by patients with epilepsy, its safety and efficacy profile is not well understood and there is no consensus on what protocol is most effective. The benefit of rTMS in the ICU is that it does not interfere with the functioning of other ICU equipment. The lack of controlled technical expertise, clinical trials, and poor insurance coverage contributes to its poor establishment as a potential treatment. Nonetheless, rTMS may be both clinically and cost-effective in the treatment of patients with SRSE, without the side effects of traditional AEDs.

#### c. Vagus Nerve Stimulation

Vagus nerve stimulation (VNS) is not widely used but was shown to be effective in a few cases of SE seen in children [153,154]. There are also reports which show effective treatment of SE in adults [155,156]. In all these cases, patients were already on multiple anticonvulsants which complicates the effects of VNS. Systemic review was conducted to evaluate the effectiveness of VNS in RSE and SRSE. In this review, 45 patients were identified, of which 38 received acute implantations of VNS in RSE/SRSE. Five cases had VNS implantation for epilepsia partialis continua, one for refractory electrical status epilepticus in sleep, and one for acute encephalitis with refractory repetitive focal seizures. Emergent VNS implantation resulted in seizure cessation in 74% of acute cases. Positive outcomes occurred in 82% of cases. However, more studies are needed to understand the effect of VNS in refractory cases [157].

#### d. Deep Brain Stimulation

There is evidence that deep brain stimulation of the anterior and centromedian nuclei of the thalamus, subthalamic nucleus, striatum, globus pallidus and cerebellum are effective in controlling seizures [158-161]. There is one study in which stimulation of the anterior thalamic nucleus inhibited SE in experimental pilocarpine rat models [161], but the use of deep brain stimulation in the treatment of SRSE in humans is underwhelming.

### 5. Emergent Resective Epilepsy Surgery

Emergency surgical resection has been used as a last option for the treatment of SRSE in selected cases where there is a clear radiological lesion or evidence of focal onset ictal electrographic focus. The common surgical procedure is focal resection in cases of defined lesion. Multiple subpial transactions have been reported in five patients in combination with lesion resection in four patients, but the outcome of emergency resective epilepsy surgery is not good in most cases [162-164].

### 6. Ketogenic Diet

The ketogenic diet (KD) was first introduced as a treatment to epilepsy in the 1920’s and is still the cornerstone for the treatment of severe childhood encephalopathies. Emergency use of a ketogenic diet has also been reported in 20 cases of SE, most of which were pediatric in nature.

In a case series published by Franc ¸ois et al., six children with SRSE responded well to emergent use of KD [165]. Another case series published by Nabbout et al. included nine cases of SRSE and also found favorable outcomes [166]. Additionally, published case reports of four adult epilepsy patients with prolonged SE similarly responded well to KD [167-169].

The ketogenic diet, as an additive or alternate treatment, appears to be safe and reasonably efficient for adults with RSE and SRSE. However, the diet regimen requires professional dietician help, and it is important that infusions which include carbohydrates (drug carriers such as propylene glycol) are avoided. Moreover, KD’s use is challenging in an ICU setting as it takes about 2-3 days to reach ketosis [5].

### 7. Pyridoxine Infusion

Pyridoxine (Vitamin B6) is a coenzyme for apoenzymes such as glutamate decarboxylase and GABA transaminase, each of which are required for the production of GABA in the brain [170]. As a result of mutation in the metabolism of pyridoxine, patients can present with SE and therefore need to be supplied with intravenous pyridoxine [171]. Shorvon and Fersili in their review address that pyridoxine infusion can be administered to patients without a deficit in pyridoxine metabolism and successfully suppress seizures. It is a treatment of choice for children suffering from SRSE due to its minimal side effects [24]. The suggested effective dose is 180-600 mg/day [172]. In a study, intravenous pyridoxine (100-300?mg) was given to 12 cases with SRSE but none showed positive response. When pyridoxal-5-phosphate (30?mg/kg/day), which is also effective for patients suffering from pyridoxine dependent epilepsy, was added in 6 patients, complete seizure control in 2 patients who had neonatal onset epilepsy was achieved [173].

### 8. Cerebrospinal Fluid Drainage

Cerebrospinal fluid (CSF) drainage was utilized in the early 20th century as a method to terminate SE. The benefit of CSF drainage is well noted; however, the mechanism is still unclear. Shorvon and Ferlisi hypothesize that this could be due to the removal of inflammatory or other noxious substances, a reflex autonomic effect, or an effect on intracerebral pressure [4]. Due to the advancement of antiepileptic drugs and other therapies, this treatment is quite antiquated. A case study focusing on a 43-year-old woman with a history of drug-resistant focal and secondary generalized tonic-clonic seizures presenting with SE showed that CSF drainage was performed as a last resort after the drug treatments failed. Approximately 25 ml CSF was removed, and 70 mL of air was administered. During the procedure, the status was terminated. However, the effect was transient, and recurrence of SE occurred after one week. Repeating the procedure had no effect on the patient [174]. Despite the fact that the treatment had only a transient effect, it shows promise in treating SRSE when surgery, current therapies, and first and second drugs are not an option.

### 9. Magnesium Infusion

Magnesium sulphate is well-known for its efficacy in preventing eclamptic seizures and has been used for this purpose since the early 20^th^ century. The mechanism is unclear, but it is believed to act by inhibiting the N-methyl-D-aspartate (NMDA) receptor [175]. There have been a few case reports that have documented a successful outcome of MgSO4 infusion in the management of SRSE [176]. The suggested regimen is an initial bolus of 4 g followed by continuous infusion at 2-6 g/h, with the target plasma level being 3.5 mmol/L [177]. Although there is a lack of evidence displaying its effectiveness in SRSE, MgSO4 infusion is relatively safe and is therefore a recommended alternative in cases that persist despite treatment with first and second-line agents. At high doses, potential adverse effects include hypotension, arrhythmia, and neuromuscular block [4]. Infusion is contraindicated in cases of severe kidney failure, defined by a creatinine clearance below 30 ml/min [178].

## Prognosis of Super-Refractory Status Epilepticus

The prognosis of SRSE after treatment highly depends on the etiology of SE [5]. Long-term mortality of SRSE is approximately 30-50%. Nelson and colleagues reported that SRSE patients had longer stays in both the neurologic ICU and in the hospital. They were also more likely to be functionally dependent at hospital discharge compared to RSE patients [179]. SRSE can lead to poor outcomes, but it is unclear whether diagnosing and treating seizures affects the outcome because most cases of SRSE are due to an underlying severe brain injury [180]. Worse outcomes have been seen when patients were older than 60 years and when treatment was performed in smaller hospitals. The presence of comorbidities and SE complications are other factors which contribute to worse outcomes [181]. Short term mortality is highest when SE results from an acute brain injury such as stroke, anoxia, or infection. The mortality is low with SE arising from tumors, alcohol, trauma, or other drugs. The lowest mortality occurs when the etiology is due to an exacerbation such as fever, sleep deprivation, or an intercurrent illness [2]. When SRSE relapses when aggressive therapy is withdrawn, there is a danger of a prolonged ICU course (similar to RSE), and it may be necessary to intervene and change the treatment plan [182].

**Table 3:** STESS Scoring System.

Status Epilepticus Severity Score (STESS)		
Factor	Categories	Score
Age	< 65 years ≥ 65 years	0 2
Worst seizure type	Simple, complex, or absence Generalized convulsive Nonconvulsive status epilepticus in coma	0 1 2
Level of consciousness	Alert or somnolent Stuporous or comatose	0 1
History of seizures	Yes No	0 1

The Status Epilepticus Severity Score (STESS), a scoring system to predict outcome in SE, has been recently developed (Table 3). It is based on four factors which comprise age, seizure type, level of consciousness, and history of seizures [183]. A study by Rossetti and associates found that STESS was a predictor of survival and ability to achieve baseline clinical condition [183]. Hence, patients who had favorable STESS scores typically appeared to survive regardless of whether they received coma induction during their treatment [183]. The END-IT score, an acronym which stands for the score’s components, including encephalitis, nonconvulsive status epilepticus, diazepam resistance, image abnormalities, and tracheal intubation, was also created recently as an outcome prediction tool. The independent predictors of unfavorable outcomes at three months post-discharge include encephalitis, nonconvulsive SE, diazepam resistance, imaging abnormalities, and intubation. The END-IT score is calculated by giving a point to each category (Table 4). A higher score means a higher chance of unfavorable outcomes. For example, an END-IT score of 3 or greater seemed to be the cutoff point for predicting a negative outcome [184].

Patients with SRSE can survive in an anesthetic coma for months, given they do not have severe systemic comorbidities, and have a chance of a good outcome if the seizures are controlled. Unfortunately, information about long-term cognitive outcomes is limited. However, a portion of patients with SRSE might return to work and can improve gradually over time [185] Moreover, patients with SRSE may develop brain atrophy and cerebral microbleeds, but the incidence, cause, and functional implications of these sequelae are unknown. Regarding long-term seizure risk after SRSE, many patients may develop medically refractory epilepsy, but recurrence of SRSE is uncommon [185].

**Table 4:** END-IT Scoring System.

END-IT Score		
Factor	Categories	Score
Encephalitis	No Yes	0 1
Nonconvulsive status epilepticus	No Yes	0 1
Diazepam resistance	No Yes	0 1
Imaging abnormalities	No lesion Unilateral lesions Bilateral lesions/diffuse cerebral edema	0 1 2
Tracheal intubation	No Yes	0 1

## Discussion

Super-refractory status epilepticus is a neurological emergency with a guarded prognosis. Prognosis and future outcomes depend on the successful and timely management of the condition.

Status epilepticus is defined as super-refractory when seizures continue clinically or electrographically on continuous video EEG monitoring for more than 24 hours despite induction of pharmacologically induced coma with highly sedating anesthetic agents, or recurrence of seizures when weaning off from anesthetics [4,5].

Management of super-refractory status epilepticus requires monitoring in the intensive care unit with anesthetics and anticonvulsants. Benzodiazepines remain the first-line agents in status epilepticus and require midazolam with assisted mechanical ventilation with continued seizure activity. Second-line anticonvulsants may follow. The commonly used second-line anticonvulsants are phenytoin, phenobarbital, and levetiracetam. There is limited evidence that one is superior to the other when compared in clinical trials. The choice depends on the existing comorbidities in each patient. Levetiracetam is avoided in patients with renal failure, and phenytoin and phenobarbital are avoided in patients with hepatic dysfunction. Super-refractory status epilepticus warrants the need for third-line agents and if needed. Additional options including hypothermia, electroconvulsive therapy, and infusions of pyridoxine and magnesium. Lesionectomy and resective epilepsy surgery are indicated in the case of an identified lesion being the epileptogenic focus including tumors, cavernous malformations, medial temporal sclerosis, or in cases of herpes encephalitis with a predilection for the temporal lobe. CSF drainage is also used in limited cases. Neuromodulation techniques are used in select cases including transcranial magnetic stimulation, vagus nerve stimulation, and deep brain stimulation [4,5].

The main limitations to therapy for status epilepticus are the duration of the stay in the intensive care unit and the withdrawal from anesthetics. This is a challenge due to their highly sedating properties and subsequent need to monitor for recurrence. The use of midazolam, phenobarbital, and propofol is successful in the resolution of super-refractory status epilepticus. The primary focus is to monitor the patients and prevent the common recurrence of seizures. This may require the addition of multiple anticonvulsants, monitoring their side-effects, maximizing their dosages, and if needed, using additional therapies such as resective surgeries in cases with guarded prognosis [5].

Intensive seizure monitoring in the ICU remains the cornerstone of management and is the standard of care to monitor seizures, brief ictal rhythmic discharges, isolated seizures, or recurrence of status epilepticus. The goal is to achieve a balanced approach in which the risk of recurrence is minimized. However, complete resolution of seizures should not be expected. Patients with isolated or brief seizures can be treated in the non-ICU environment and subsequently as out-patients. Prolonged ICU stay can cause infections including urinary tract infections, ventilator-associated pneumonia, deep venous thrombosis, critical illness myopathies and neuropathies, ICU delirium, and blood infections which further complicates the management of the primary condition [5].

Another challenge in the treatment of super-refractory status epilepticus is that there is limited evidence regarding alternative available approaches in the management of this condition as benzodiazepines remain the first-line treatment [35].

There have been strides in basic science research over the last two decades which show that seizures tend to endure as a result of an imbalance between excessive and insufficient neuronal excitation, or due to a defect in the neuronal inhibition systems [185,186]. The common inhibitory neurotransmitter, γ-Aminobutyric acid (GABA), inhibits neurons from excess excitation through the activation of the GABA receptors, and the excitatory neurotransmitter, glutamate, controls excitation through the N-methyl-S-aspartate (NMDA) receptor [187]. SE can become self-sustaining with neuronal damage and pharmacoresistance, progressing to RSE or SRSE [188]. A process known as receptor trafficking can occur in SRSE in which there is an increase in the number of glutaminergic receptors at the cell surface and a decrease in GABA receptors, resulting in decreased GABAergic activity [189]. Besides receptor trafficking, there could be a plethora of other mechanisms contributing to the existence of SE, RSE, and SRSE which can be utilized as potential therapeutic targets. Some of the reported mechanisms include mitochondrial failure or insufficiency, inflammatory processes that disrupt the blood-brain barrier, deficiency of cofactors and vitamins, control mechanisms of calcium flux, and genetic alterations [190, 191-194]. Pathophysiological processes are related to the processes of protein phosphorylation and activation channels in neurons. Antiepileptic drugs available carry out their activity in the transport of intracellular vesicles, or through the activity of inhibitory receptors [195].

Studies have also shown that neuroinflammation and oxidative stress occur rapidly in the brain during SE and have the potential to continue to persist, creating the acute and long-term sequelae of SE [196]. Reactive oxygen species (ROS) have been indicated to be a mediator in neuronal injury and are considered to produce proinflammatory cytokines during epilepsy [197]. A potential biomarker for epilepsy known as high mobility group box-1 (HMGB1) has recently emerged. HMGB1 activates macrophages, endothelial cells, and other immune response pathways and thus elevates pro-inflammatory protein levels [198]. Studies have shown that there is an increase in the level of HMGB1 within 3-4h after drug-resistant epilepsy [199]. Neuroinflammation and oxidative stress markers are measurable in peripheral blood and by neuroimaging [196]. These findings show a promising avenue for developing prognostic and predictive mechanistic biomarkers in people exposed to status epilepticus.

MicroRNAs (miRNAs) have been indicated to play a role in epileptogenesis [200]. High levels of miRNA-23a, miRNA-34a, miRNA-132, miRNA-146a, in particular, were frequently detected. Additionally, increased levels of miRNA-21, miRNA-29a, miRNA-132, identified to be regulated by p53, were noted to be involved in episodes of seizures [201]. In particular, Wang et al. reported that miRNA-451 has the potential to be a biomarker for refractory epilepticus [202]. Specific miRNAs can be investigated to see if they are potential biomarkers for different types of epilepsies such as SE, RSE, and SRSE.

Lastly, translational research involving animal-to-human studies which identify potential biomarkers as specific molecular targets can help us to develop novel therapeutic agents. Once identified in animal models, these biomarkers can then be tested in human subjects for efficacy by conducting multicenter clinical trials.

## Conclusion

Super-refractory status epilepticus remains a common neurological emergency encountered in intensive care units throughout the world and remains a significant management challenge. Morbidity and mortality of super-refractory status epilepticus depend on initial effective treatment by induction of pharmacological coma to abort refractory seizures. Clinicians and patients would benefit from a comprehensive meta-analysis of prognostic factors and studies directed to management and their outcomes. There continues to be a need for large randomized multicenter drug trials to test the efficacy of new treatment strategies in this refractory condition.
